# Resistance of *Trichoplusia ni* to *Bacillus thuringiensis* Toxin Cry1Ac Is Independent of Alteration of the Cadherin-Like Receptor for Cry Toxins

**DOI:** 10.1371/journal.pone.0035991

**Published:** 2012-05-14

**Authors:** Xin Zhang, Kasorn Tiewsiri, Wendy Kain, Lihua Huang, Ping Wang

**Affiliations:** Department of Entomology, Cornell University, New York State Agricultural Experiment Station, Geneva, New York, United States of America; Ghent University, Belgium

## Abstract

Alteration of binding sites for *Bacillus thuringiensis* (Bt) toxins in insect midgut is the major mechanism of high-level resistance to Bt toxins in insects. The midgut cadherin is known to be a major binding protein for Bt Cry1A toxins and linkage of Bt-resistance to cadherin gene mutations has been identified in lepidopterans. The resistance to Bt toxin Cry1Ac evolved in greenhouse populations of *Trichoplusia ni* has been identified to be associated with the down-regulation of an aminopeptidase N (APN1) gene by a *trans*-regulatory mechanism and the resistance gene has been mapped to the locus of an ABC transporter (ABCC2) gene. However, whether cadherin is also involved with Cry1Ac-resistance in *T. ni* requires to be understood. Here we report that the Cry1Ac-resistance in *T. ni* is independent of alteration of the cadherin. The *T. ni* cadherin cDNA was cloned and the cadherin sequence showed characteristic features known to cadherins from Lepidoptera. Various *T. ni* cadherin gene alleles were identified and genetic linkage analysis of the cadherin alleles with Cry1Ac-resistance showed no association of the cadherin gene with the Cry1Ac-resistance in *T. ni*. Analysis of cadherin transcripts showed no quantitative difference between the susceptible and Cry1Ac-resistant *T. ni* larvae. Quantitative proteomic analysis of midgut BBMV proteins by iTRAQ-2D-LC-MS/MS determined that there was no quantitative difference in cadherin content between the susceptible and the resistant larvae and the cadherin only accounted for 0.0014% (mol%) of the midgut BBMV proteins, which is 1/300 of APN1 in molar ratio. The cadherin from both the susceptible and resistant larvae showed as a 200-kDa Cry1Ac-binding protein by toxin overlay binding analysis, and nano-LC-MS/MS analysis of the 200-kDa cadherin determined that there is no quantitative difference between the susceptible and resistant larvae. Results from this study indicate that the Cry1Ac-resistance in *T. ni* is independent of cadherin alteration.

## Introduction

The soil bacterium *Bacillus thuringiensis* (Bt) is the most successfully used microbial insect control agent in agriculture and public health [1], [2]. Since 1996, transgenic crops engineered with insecticidal Bt toxin genes (Bt-crops) to confer insect resistance have been rapidly adopted worldwide. In 2010, Bt-crops were planted on close to 60 million hectares [3]. However, development of resistance to Bt toxins in insect populations threatens the sustainable application of both sprayable Bt-based biopesticides and Bt-crops for insect control. The potential for development of insect resistance to Bt toxins has been widely demonstrated by successful establishment of various Bt-resistant insect populations through selection with Bt toxins under laboratory conditions [4], [5]. In agricultural settings, cases of insect resistance to Bt biopesticides and Bt-crops have been reported in six lepidopteran species [6]–[10]. To achieve continuing success in application of Bt-based technologies for insect pest control, it is crucially important to understand the molecular genetics of Bt resistance evolved in insect populations in agricultural systems, which is currently unclear for any case of field- or greenhouse-evolved Bt-resistance.

The pathways of Bt pathogenesis in insects are complex [11], [12]. In insect midgut, Bt Cry protoxins are activated through proteolytic cleavages by insect digestive proteases. The activated toxins pass through the midgut peritrophic membrane, a protective midgut lining, into the ecto-peritrophic space of the midgut, where the toxins reach the target site, midgut brush border membrane. At the midgut brush border membrane, Cry toxins interact with specific receptors, which are not fully understood at present [13]–[15], and insert into the membrane in an oligomeric form to form lytic pores, leading to cell lysis [16], [17]. Alternatively, binding of Cry toxins with the midgut cadherin-like protein has been suggested to activate a cellular signaling pathway leading to cell death, based on studies in cell culture [18], [19]. An alteration of any event in the complex Bt pathogenesis pathway may potentially lead to resistance to Bt toxins in insects. Therefore, mechanisms of Bt resistance in insects can be diverse [14], [20], [21]. Reported mechanisms of Bt resistance include alteration of Cry toxin solubilization and midgut proteases, decreased permeability of the peritrophic membrane to Cry toxins, heightened insect immune response, increased sequestering of the toxins in the midgut by enhanced esterase production and, more importantly, reduced binding of the toxins to the midgut brush border membrane [1], [22]–[27]. “Mode 1” type resistance is the most common high-level resistance to Bt Cry1A toxins in insects, which is characterized by a very high level of resistance to at least one Cry1A toxin, recessive inheritance, reduced binding of at least one Cry1A toxin to the midgut brush border membrane and negligible cross-resistance to Cry1C toxins [27]. The “Mode 1” type resistance in laboratory-selected strains of three cotton pests, *Heliothis virescens*, *Pectinophora gossypiella* and *Helicoverpa armigera*, has been identified to be linked with mutations of the midgut cadherin gene [28]–[30]. The linkage of cadherin gene mutations with “Mode 1” type resistance can be exploited to develop molecular tools for detection of the resistance alleles, which is particularly useful for monitoring development of Bt resistance which is typically recessive in inheritance in insect populations in the field [31]–[33]. However, it has become evident that insect resistance to Bt toxins selected under laboratory conditions does not necessarily share the same genetic mechanism with that selected in agricultural situations [34], [35]. The field-evolved resistance to Bt in diamondback moth, *Plutella xylostella*, exhibits typical “Mode 1” type resistance, but is not genetically linked with the cadherin gene [36]. Instead, the resistance has been mapped to the genetic locus of an ABC transporter (ABCC2) gene [37]. Similarly, the “Mode 1” type resistance evolved in greenhouse populations of *T. ni* has been recently identified to be associated with down-regulation of an aminopeptidases N (APN1) by a *trans*-regulatory mechanism [35]. Although the resistance-conferring *trans*-acting gene remains to be functionally identified, the resistance gene has been genetically mapped to the ABCC2 gene locus [37]. Whether the cadherin is also involved in the Cry1Ac-resistance in either *P. xylostella* or *T. ni* requires to be understood. Therefore, more studies are required on the role of midgut cadherin in Bt-resistance in a broad range of cases of Bt-resistance to understand the extent of association of cadherin mediated Bt resistance evolved in insect populations in agricultural systems. In this study, the potential involvement of the midgut cadherin in resistance to Bt toxin Cry1Ac in *T. ni* was comprehensively examined from genetic linkage of various cadherin gene alleles with the resistance to the expression level of the cadherin protein and its binding to the toxin. Results from this study indicate that Bt-resistance evolved in the greenhouse populations of *T. ni* is independent of alteration of the midgut cadherin, which is different from the cadherin mutation-associated genetic basis of “Mode 1” type resistance.

## Materials and Methods

### Insect Strains

A highly inbred laboratory strain of *T. ni* (named Cornell strain), which has never been exposed to Bt in the laboratory [38], was used as the susceptible strain. Cry1Ac-resistant *T. ni* used in this study was the GLEN-Cry1Ac-BCS strain [39] when it had been backcrossed with the susceptible Cornell strain 4 times (named GLEN-Cry1Ac-BCS4) and 8 times (named GLEN-Cry1Ac-BCS8). The larvae were reared on a high wheat germ diet [40] at 27±1°C, 50% RH, and with a 16 h-light–8h-dark photoperiod. Adults were maintained under the same temperature and light conditions at a RH of 60% and supplied with 10% sucrose solution.

### Preparation of Bt Cry1Ac toxin

Cry1Ac protoxin crystals were prepared from *Bt kurstaki* strain HD-73 (obtained from the *Bacillus* Genetic Stock Center, http://www.bgsc.org/) and solubilized in 50 mM Na_2_CO_3_ buffer (pH 9.5) with 50 mM EDTA and 5% β-mercaptoethanol, followed by centrifugation to remove insoluble materials, and finally the protoxin was precipitated with sodium acetate (pH 4.5) to remove unprecipitated impurities as described by Kain et al. [38]. The Cry1Ac protoxin was stored at −20°C and used for larval feeding assays.

Activated Cry1Ac toxin was prepared by treatment of the protoxin with TPCK-treated trypsin (Sigma, St Louis, MO) at a ratio of 1∶20 (w/w) in 50 mM Na_2_CO_3_ (pH 10.0) at 37°C for 1 to 16 h and proteolytic activation was examined by SDS-PAGE analysis. The activated Cry1Ac toxin was purified by anion-exchange chromatography on a UNO Q column (Bio-Rad Laboratories, Hercules, CA) eluted with a linear gradient from 0 to 1.0 M NaCl in 20 mM Na_2_CO_3_ (pH 10.0) and fractions containing purified toxin identified by SDS-PAGE analysis were pooled and stored at −20°C.

### Cloning of *T. ni* Cadherin cDNA

Based on the cadherin sequences from *Manduca sexta* (AAG37912), *Heliothis virescens* (AAK85198), *Helicoverpa armigera* (ABF69362), *Bombyx mori* (AB041510) and *Lymantria dispar* (AAL26896), a pair of degenerate primers, 5′-ATHAAYTGGAAYGAYGAR-3′ and 5′-ACRTTYTCYTCNAC-3′, were designed and used for PCR amplification of a fragment of cadherin cDNA from a *T. ni* midgut cDNA library [41]. The PCR reaction mix contained 0.2 µM of primers, 0.2 mM dNTPs, 1 µl of the cDNA library suspension (1×10^7^ plaques/µl) and 2.5 units of *Taq* DNA polymerase (New England Biolabs, Beverly, MA) in a 50 µl reaction. The PCR was performed under the following conditions for 35 cycles: 94°C×30 s, 45°C×30 s, and 72°C×1 min. The amplified PCR fragment was purified by excision of the DNA band after agarose gel electrophoresis, followed by recovery of the DNA fragment using the QIAEX®II Gel Extraction Kit (Qiagen, Valencia, CA), and cloned into the pGEM-T vector (Promega, Madison, WI). The cDNA insert was sequenced, followed by a BLAST sequence similarity search to confirm the correct amplification of the *T. ni* cadherin cDNA fragment. This *T. ni* cadherin cDNA fragment (383 bp) was labeled with digoxigenin using a DIG High Prime DNA Labeling and Detection kit (Roche Applied Science, Indianapolis, IN) following the instructions provided by the manufacturer and used as a probe for screening of the *T. ni* midgut cDNA library to identify *T. ni* cadherin cDNA clones from the library. Positive phage clones were isolated and then subjected to an in vivo plasmid excision procedure to recover the pBluescript plasmids using the Uni-ZAP XR Vector System (Stratagene, La Jolla, CA). The 5′ end fragment of the cadherin cDNA, which was missing in the cDNA clones obtained from the cDNA library screening, was amplified by PCR from the *T. ni* midgut cDNA library using the primer T3, which is located upstream of the cDNA insert in the lambda vector, and a cadherin specific primer (5′-GCCTCGTAGTCCTGCTTATTAGTG-3′) designed based on the sequence from the partial *T. ni* cadherin cDNA clones. A PCR fragment of about 700 bp, including the 5′-end fragment of the cadherin cDNA and a fragment of the lambda vector, was cloned into the pGEM-T easy vector (Promega) and sequenced.

Sequence analysis was performed using the Lasergene software package (DNAStar, Madison, WI). Secretory signal sequence was predicted using SignalP (http://www.cbs.dtu.dk/services/SignalP/), and transmembrane domain searches were performed using TMHMM (http://www.cbs.dtu.dk/services/TMHMM). Protein sequence motif scan was performed using the Motif Scan tool on the Myhits web server (http://myhits.isb-sib.ch/cgi-bin/motif_scan).

### Genotyping of *T. ni* Cadherin Gene by PCR Amplification and Sequencing of Genomic DNA Fragments

Two genomic DNA fragments of the cadherin gene, covering the cDNA sequence (Genbank accession no. JF303656) regions from nucleotide position 1705 to 1856 (gDNA fragment 1), and 4911 to 5114 (gDNA fragment 2), were used to genotype the cadherin gene in *T. ni* individuals. Genomic DNA was prepared from 5^th^ instar larvae or adults using a rapid genomic DNA preparation method [35]. A genomic DNA fragment of 519 to 563 bp covering the cDNA region from 1705 to 1856 containing an intron of 367–411 bp was amplified by PCR with a primer set 5′-ACGAGCTCCCGATCTTCGA-3′ and 5′-CAGATAATCTTCAGCATTGCC-3′. A fragment of 495 bp, covering the cDNA sequence from position 4911 to 5114 and a 291 bp intron was amplified by PCR with a primer set 5′-GCGCTGCTGGGCTTCCTGT-3′ and 5′-CGCTTTGATGGTCTCGTTC-3′. The amplification reactions (25 µl) contained 0.5 µl of genomic DNA template, 0.2 µM of each primer, 0.2 µM of dNTPs, 2.5 µl 10× PCR buffer, and 1 U of *Taq* polymerase. Reactions were performed for 40 cycles of 30 s at 94°C, 30 s at 55°C, and 40 s at 72°C followed by a final extension at 72°C for 10 min. The PCR amplified fragments were sequenced to determine the cadherin genotypes.

### Genetic Linkage Analysis of Cadherin Gene with Cry1Ac Resistance

Single-pair crosses using males from the susceptible Cornell strain and females from the GLEN-Cry1Ac-BCS4 strain, which contained multiple cadherin gene alleles, were prepared to generate F_1_ families. Once eggs were collected, the adults were subjected to genotyping of their cadherin gene alleles as described above. F_1_ families from parents with different cadherin gene alleles were selected and maintained on artificial diet to pupation. Thirty females and 30 males from each selected family were placed in a cage to allow intra-crossing to generate F_2_ progenies. Five hundred eighty to six hundred thirty neonates from each F_2_ family were treated with 5 µg Cry1Ac/cm^2^ for 8 days using a diet overlay method [38] to eliminate homozygous and heterozygous susceptible individuals. Larval mortalities were recorded and survivors were used for genotyping of the cadherin gene after being reared to the 5th instar on diet without Cry1Ac. Larvae from each F_2_ family reared to the 5th instar on diet without exposure to Cry1Ac were used as non-Bt-selected controls. Thirty Cry1Ac-selected and 30 non-selected F_2_ larvae from each family were randomly chosen for genotyping of the cadherin gene alleles as described above to examine the genetic linkage of the cadherin gene with the resistance by analyzing the segregation of the cadherin gene alleles with resistance to Cry1Ac.

### Quantitative Real-time RT-PCR Analysis

Mid-fifth instar larvae from the Cornell and GLEN-Cry1Ac-BCS8 strains were dissected in cold Rinaldini’s solution [42] to isolate the midgut tissue. PMs with food contents and other attached tissues were quickly removed and the isolated midgut tissue was rinsed with cold Rinaldini’s solution, and then individually stored in the RNA*later* solution (Ambion, Austin, TX) at −20°C. Total RNA from an individual midgut was isolated using the RNeasy® Mini Kit (Qiagen Inc., Chatsworth, CA) coupled with an on-column DNase digestion procedure to ensure absence of genomic DNA contamination in the RNA preparations. cDNAs were prepared from the total RNA preparations using the ImProm-II™ Reverse Transcription System (Promega) following the instructions provided by the manufacturer for quantitative real-time PCR analysis.

Real-time PCR samples were prepared in the iQ™ SYBR® Green Supermix (Bio-Rad Laboratories) with the cadherin gene specific primers 5′-GCGCTGCTGGGCTTCCTGT-3′and 5′-CGCTTTGATGGTCTCGTTC-3′. Real-time PCR reactions were performed on the IQ5 real-time PCR detection system (Bio-Rad) with a 2 min heating at 95°C, followed by 40 cycles of amplification at 95°C for 10 s, 58°C for 30 s and 72°C for 30 s. The *T. ni* β-actin gene (Genbank accession no. JF303662) was used as a house-keeping gene for internal control with primers 5′-GTTGCTGCGTTGGTAGTAGACA-3′ and 5′-TCCCAGTTGGTGACGATGC-3′. The level of cadherin gene expression was defined as relative level of the cadherin gene transcript to the actin gene transcript determined by the real-time RT-PCR analysis. Three larvae from each strain were analyzed with three technical replications for each sample.

### Preparation of Midgut Brush Border Membrane Vesicles (BBMVs)

Midgut BBMVs were prepared following the method developed by Wolfersberger et al [43]. Mid-fifth instar larvae from the Cornell strain and GLEN-Cry1Ac-BCS8 strain were immobilized on ice and dissected in cold dissection buffer (17 mM Tris-HCl, pH 7.5, 5 mM EGTA, 300 mM mannitol, 1 mM PMSF) to isolate the midgut epithelium. The midgut epithelial tissue was homogenized in an equal volume of ice-cold 24 mM MgCl_2_, then incubated on ice for 15 min, followed by centrifugation at 2,500×g at 4°C for 15 min to collect the supernatant. The pellet from the centrifugation was resuspended in ice-cold dissection buffer in 0.5 volume of the initial homogenate and then the BBMV extraction procedure was repeated as described above. The supernatants collected from the two extractions were combined and the BBMVs were precipitated by centrifugation at 30,000×g at 4°C for 1 h and stored at −80°C. The protein concentration of the BBMV preparations was determined using the Bradford method [44]. Enzymatic activities of the brush border membrane marker enzymes alkaline phosphatase and aminopeptidase in the BBMV preparations and in the initial midgut tissue homogenates were determined as described by Jurat-Fuentes and Adang [45] to evaluate the enrichment of brush border membranes in the BBMV preparations. The enrichment of the two marker enzyme activities typically ranged 5–6 and 7–10 fold, respectively.

### Quantitative Proteomic Analysis of midgut BBMV Proteins

Isobaric tagging for relative and absolute quantitation (iTRAQ) technique was used for quantitative comparative analysis of the cadherin content in the midgut BBMVs of the susceptible and resistant larvae as reported by Tiewsiri and Wang [35]. Midgut BBMV containing 5 mg proteins was solubilized in 0.5 ml of 0.5 M HEPES (pH 7.4) with 5 mM EGTA, 0.3 M mannitol and 1% SDS. Solubilized BBMV proteins were reduced with 5 mM tris-(2-carboxyethyl)-phosphine at 37°C for 1 h and treated with 8 mM methyl methanethiosulfonate at room temperature for 10 min to block the thiol groups. The proteins were then digested with sequencing grade modified trypsin at 37°C for 16 h and the resulting tryptic peptides were labeled with the iTRAQ™ reagent (Applied Biosystems, Foster City, CA) using the protocols provided by the manufacturer. Each sample was separately labeled with two different ion reporter reagents for technical replications. The sample from the Cornell strain was labeled with reporter ion tags 114 and 116, and the sample from the GLEN-Cry1Ac-BCS strain with reporter ion tags 115 and 117. The labeled samples were combined and fractionated by OFFGEL IEF electrophoresis using an Agilent 3100 OFFGEL Fractionator (Agilent, Santa Clara, CA) with Immobiline™ DryStrip pH 3–10 (24 cm) (GE Healthcare, Piscataway, NJ). The fractions collected from the OFFGEL Fractionator were pooled into 10 fractions and acidified with 1% trifluoroacetic acid, desalted by solid-phase extraction with a Sep-Pak® C18 cartridge (Waters, Milford, MA), dried and finally reconstituted in 2% acetonitrile in 0.5% formic acid for subsequent nano-LC-MS/MS analysis.

Nano-LC-MS/MS analysis of the tryptic peptides labeled with iTRAQ tags was performed using the LTQ Orbitrap Velos mass spectrometer (Thermo-Fisher Scientific, San Jose, CA) with high-energy collisional dissociation at the Proteomics and Mass Spectrometry Core Facility of Cornell University (Ithaca, NY). The LTQ Orbitrap Velos was interfaced with an UltiMate® 3000 Proteomics MDLC system (Dionex, Sunnyvale, CA) for nano-LC. Peptide samples obtained from above were injected onto a PepMap C18 trap column (5 µm, 300 µm×5 mm) (Dionex) for on-line desalting and then separated on a PepMap C-18 RP nano column (3 µm, 75 µm×15 cm) (Dionex). The eluted peptide fractions from the PepMap C-18 RP nano column were analyzed in the LTQ Orbitrap Velos through nano ion source with a 10-µm analyte emitter (New Objective, Woburn, MA). Data were acquired with the Xcalibur 2.1 software (Thermo-Fisher Scientific). The MS/MS raw spectra obtained were processed with the software Proteome Discoverer 1.1 (Thermo-Scientific), followed by subsequent database search using the software Mascot Deamon version 2.2.04 (Matrix Science, Boston, MA) with a *T. ni* protein sequence database containing 15,536 sequence entries generated by combining 12,457 sequences (including 12,294 ESTs) downloaded from the Genbank (http://www.ncbi.nlm.nih.gov/genbank/) on Nov. 20, 2009 and 3,079 sequences (including 2,992 ESTs) generated in the authors’ laboratory. For protein identification and iTRAQ quantitative data processing, peptide mass tolerance and fragment mass tolerance values were set at 10 ppm and 30 mDa, respectively. The significance threshold was set at the 95% confidence interval and only those peptides that passed this filter were used for protein identifications. Furthermore, only the identified proteins containing at least two peptides with a *p* value <0.001 determined by Mascot probability analysis were selected for further analysis. Intensities of the reporter ions (114, 115, 116 and 117) from iTRAQ tags upon fragmentation were used for protein quantification, and the relative protein ratios were normalized at the median ratio.

Technical variations of the iTRAQ analysis were evaluated with the data from the two technical replicates (tag 114 and tag 116 for the susceptible Cornell strain, and tag 115 and tag 117 for the GLEN-Cry1Ac-BCS8 strain) for each BBMV protein sample included in the iTRAQ runs to ensure the quality of the analysis. The difference between the two technical replicates in protein ratio, |log_2_(117/115)| and |log_2_(116/114)|, that covered 95% of the identified proteins was defined as internal/technical error [35], [46]. Biological variation was determined with the data from two independently prepared sets of midgut BBMV samples. The difference in protein ratio between the two sample sets [Δlog_2_ ratio  =  |log_2_ ratio(sample set1) −log_2_ ratio(sample set2)|] covering 90% or 95% of the identified proteins was defined as biological variation to evaluate the significance of quantitative difference between the proteins [35], [46].

### Toxin Overlay Binding Assay and Cry1Ac Binding Protein Identification by Nano-LC-MS/MS

Binding of Cry1Ac to *T. ni* larval midgut BBMV proteins was analyzed by a toxin overlay binding assay as described by Bravo et al. [47]. Twenty µg of BBMV proteins prepared from the Cornell strain and GLEN-Cry1Ac-BCS8 strain solubilized in SDS-PAGE sample buffer were separated by 10% SDS-PAGE and transferred onto the polyvinylidene difluoride membrane Immobilon-P (Millipore, Billerica, MA). After incubation in 5% nonfat milk with 0.5% Tween 20 in PBS (pH 7.4) at room temperature for 1 h, the membrane was incubated with 10 nM purified active Cry1Ac toxin in PBS-T (PBS with 0.05% Tween 20) at room temperature for 2 h. Unbound toxin was removed by washing the membrane 3 times with PBS-T. The binding of Cry1Ac to the BBMV proteins on the membrane blot was detected with rabbit antibodies specific to Cry1Ac followed by the secondary anti-rabbit IgG antibody conjugated with alkaline phosphatase (Sigma). The positive reaction was finally visualized with a colorimetric reaction with nitroblue tetrazolium/bromochloroindolyl phosphate.

A duplicate set of BBMV protein samples from the Cornell strain and the GLEN-Cry1Ac-BCS8 strain were separated on the same SDS-PAGE gel as described above. The gel slices from the susceptible and resistant strains corresponding to the positively stained band at 200 kDa on the membrane blot from the toxin overlay binding assay were excised from the gel and processed for in-gel digestion with trypsin [48], followed by protein identification by nano-LC-MS/MS as described above. The quantity of cadherin in the excised gel slice was estimated by the exponentially modified protein abundance index (emPAI) of the cadherin identified by nano-LC-MS/MS [49].

## Results

### cDNA Sequence of the *T. ni* Cadherin Gene

**Figure 1:**
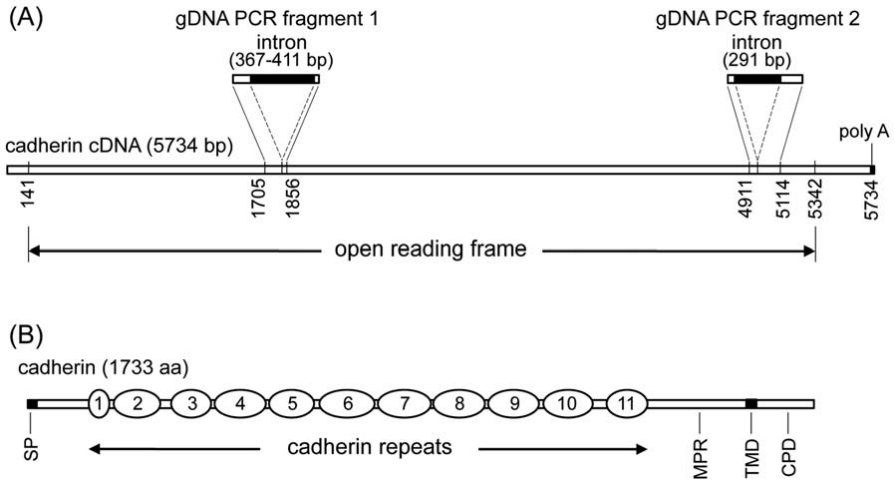
Schematic structures of *T. ni* cadherin cDNA and deduced protein sequences. (A) The cDNA (5734 bp in length) contains an open reading frame of 5202 bp from position 141 to 5342, and a poly A tail at the 3′ end. Also shown in (A) are two fragments of the genomic DNA of the cadherin gene, gDNA fragment 1 and gDNA fragment 2, amplified by PCR. gDNA PCR fragment 1 corresponds to the cDNA region from base positions 1705 to 1856 and contains an intron of 367–411 bp inserted between the cDNA base positions 1822 and 1823. gDNA PCR fragment 2 corresponds to the cDNA region from base position 4911 to 5114 and contains an intron of 291 bp inserted between cDNA base positions 4969 and 4970. (B) The deduced cadherin sequence (733 aa in length) contains a 21-aa signal peptide at the N-terminus, 11 cadherin repeats (from 1 to 11), followed by a membrane-proximal region (MPR), a transmembrane domain (TMD) of 23 amino acid residues, and a cytoplasmic domain (CPD) of 128 amino acid residues.

PCR amplification with the degenerated PCR primers designed based on known lepidopteran cadherin sequences generated a 383 bp cDNA fragment (Genbank accession no. JN849380) from the midgut cDNA library. DNA sequencing of the PCR fragment after cloning into pGEM-T vector and subsequent BLASTX search of the Genbank database indicated that the PCR fragment was a cDNA fragment of *T. ni* cadherin gene. Subsequent screening of the *T. ni* midgut cDNA library with this 383 bp fragment labeled with digoxigenin as a probe identified two cDNA clones containing a cDNA insert of 4270 bp (from nucleotide 496 to 4767, Figure S1) and 4600 bp (from nucleotide 1132 to 5732, Figure S1), respectively. A 573 bp fragment of the 5′-end of the cadherin cDNA was obtained by PCR amplification from the *T. ni* cDNA library with primer T3 and a cadherin specific primer to complete the 5,734 bp cadherin cDNA sequence (Genbank accession no. JF303656) (Figure S1 and Figure 1).

The cadherin cDNA contains an open reading frame of 5,202 bp coding for the cadherin of 1,733 amino acid residues with predicted molecular weight 194.69 kDa (Figure 1). The deduced amino acid sequence of the cadherin exhibits features characteristic of known lepidopteran cadherins [13]. It contains a 21 amino acid signal peptide at the N-terminus, 11 cadherin repeats, followed by a membrane-proximal region, a transmembrane region of 23 amino acid residues, and a cytoplasmic region of 128 amino acid residues at the C-terminus (Figure 1). Ten of the 11 cadherin repeats range from 95 to 127 aa in length identified by a search with Motif Scan using Prosite profiles with E-values <5e-5, but cadherin repeat 1 contains only 46 aa with an E-value 0.27. The protein sequence also contains seven putative N-glycosylation sites (Figure S1).

### Cadherin Gene Allele Identification in *T. ni* Strains

A genomic DNA fragment of 519 to 563 bp corresponding to the cDNA region from nucleotide 1705 to 1856 was amplified from larval genomic DNA by PCR (Figure 1). This PCR-amplified genomic DNA fragment contained an intron of 367 to 411 bp in length between the cDNA base pairs 1822 and 1823, in addition to the exon sequence between 1705 and 1856. Another 495 bp genomic DNA fragment corresponding to the cDNA region from nucleotide 4911 to 5114 was amplified by PCR. This 495 bp fragment also contained an intron of 291 bp in addition to the flanking exon regions. Both these two genomic DNA regions were used as allelic markers for genotyping of cadherin gene alleles in *T. ni* individuals to ensure reliable identification cadherin alleles. One cadherin gene allele, *cad^C1^* (Genbank accession nos. JN849384 and JN849389) was identified in the Cornell strain and four additional alleles were identified in the resistant GLEN-Cry1Ac-BCS4 strain (*cad^G1^*, *cad^G2^*, *cad^G3^* and *cad^G4^*) (Genbank acc. nos. JN849381, JN849382, JN849383, JN849385, JN849386, JN849387 and JN849388) based on the allelic variations in the two genomic DNA fragments. Genotyping of GLEN-Cry1Ac-BCS4 individuals at generations F_4_, F_19_, F_25_ and F_26_ indicated that the frequency of *cad^C1^* introgressed from the Cornell strain in the GLEN-Cry1Ac-BCS4 strains was 50%. In the further backcrossed GLEN-Cry1Ac-BCS8 strain, *cad^C1^* was found to be the only allele in 5 individuals (10 alleles in total) examined, indicating that the frequency of *cad^G^* in GLEN-Cry1Ac-BCS8 is below 0.005%.

### Cadherin Gene is not Genetically Linked with Cry1Ac Resistance

The F_2_ larvae from 4 single-pair cross families generated for the genetic linkage analysis of the cadherin gene with Cry1Ac resistance experiments exhibited a survival rate of 17% to 35% upon selection with Cry1Ac, which statistically fits the predicted survival of 25% for monogenic recessive inheritance of the Cry1Ac-resistance [38] (Table 1). Genotyping of the Cry1Ac-selected and non-selected F_2_ larvae from the 4 families showed that the *cadherin* genotype ratios *cad^G^ cad^G^*: *cad^G^ cad^C^*: *cad^C^cad^C^* in both Cry1Ac-selected and non-selected F_2_ families did not statistically deviate from the predicted ratio 1∶2∶1 for random assortment of the *cadherin* alleles inherited from their parents, demonstrating that the *cadherin* alleles from the resistant parents (*cad^G^*s) did not co-segregate with Cry1Ac-resistance and therefore are independent of the resistance to Cry1Ac (Table 2).

**Table 1:** Response of F_2_ larvae from four families of single-pair crosses to Cry1Ac selection.

Family(Genotypes of grandparents)	Number of F_2_larvae	Cry1Acselection	Survival rate^1^	Predictedsurvival rate^2^	*p*-value^3^
Family 1	580	Yes	35%	25%	0.20
*cad^G1^ cad^G1^* × *cad^C1^cad^C1^*	75	No	100%	100%	−
Family 2	630	Yes	19%	25%	0.43
*cad^G1^cad^G2^* × *cad^C1^cad^C1^*	75	No	100%	100%	−
Family 3	600	Yes	17%	25%	0.24
*cad^G2^cad^G2^* × *cad^C1^cad^C1^*	75	No	100%	100%	−
Family 4	600	Yes	17%	25%	0.23
*cad^G2^cad^G3^* × *cad^C1^cad^C1^*	75	No	100%	100%	−

1 Survival Rate (%) was corrected using the Abbott’s formula [63] with the control survival rates which were ≥95% in the bioassays.

2 Predicted survival rate 25% to selection with Cry1Ac was calculated based on inheritance of the recessive monogenic Cry1Ac-resistance trait.

3 Statistical significance was tested by *chi*-square test.

**Table 2:** Cadherin allele frequencies in F_2_ progenies from four single-pair cross families.

Family	Selection		Genotype of F_2_		# of larvae	*p-*value^1^
(male x female)	w/Cry1Ac	*cad^C^cad^C^*	*cad^C^cad^G^*	*cad^G^cad^G^*	(total)	
Family 1	no	10 *cad^C1^cad^C1^*	16 *cad^C1^cad^G1^*	4 *cad^G1^cad^G1^*	30	0.46
*cad^C1^cad^C1^* × *cad^G1^cad^G1^*	yes	10 *cad^C1^cad^C1^*	18 *cad^C1^cad^G1^*	2 *cad^G1^cad^G1^*	30	0.13
	no	7 *cad^C1^cad^C1^*	1 *cad^C1^cad^G1^*	4 *cad^G1^cad^G2^*	30	0.98
Family 2			14 *cad^C1^cad^G2^*	4 *cad^G2^cad^G2^*		
*cad^C1^cad^C1^* × *cad^G1^cad^G2^*	yes	7 *cad^C1^cad^C1^*	4 *cad^C1^cad^G1^*	4 *cad^G1^cad^G2^*	30	0.93
			12 *cad^C1^cad^G2^*	3 *cad^G2^cad^G2^*		
Family 3	no	9 *cad^C1^cad^C1^*	15 *cad^C1^cad^G2^*	6 *cad^G2^cad^G2^*	30	0.83
*cad^C1^cad^C1^* × *cad^G2^cad^G2^*	yes	7 *cad^C1^cad^C1^*	15 *cad^C1^cad^G2^*	8 *cad^G2^cad^G2^*	30	0.98
	no	6 *cad^C1^cad^C1^*	12 *cad^C1^cad^G2^*	6 *cad^G2^cad^G3^*	29	0.87
Family 4			5 *cad^C1^cad^G3^*			
*cad^C1^cad^C1^* × *cad^G2^cad^G3^*	yes	10 *cad^C1^cad^C1^*	4 *cad^C1^cad^G2^*	1 *cad^G2^cad^G2^*	25	0.32
			4 *cad^C1^cad^G3^*	5 *cad^G2^cad^G3^*		
				1 *cad^G3^cad^G3^*		

1 Statistical significance was analyzed by Chi-square test with the predicted ratios of *cad^C^cad^C^* : *cad^C^cad^G^* : *cad^G^cad^G^*  = 1∶2∶1 in the tested individuals of each treatment.

### The mRNA Level of Cadherin Gene in Cry1Ac-resistant *T. ni* Larvae is not Different from that in the Susceptible Strain

**Figure 2:**
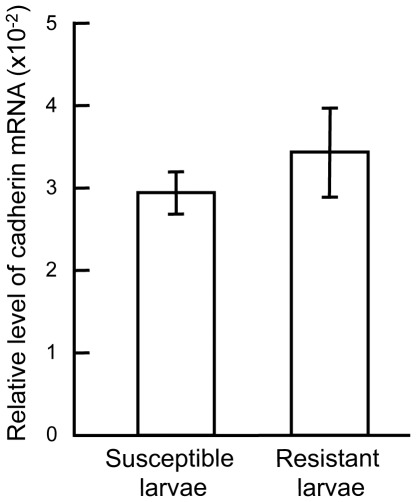
Relative levels of cadherin mRNA, normalized to the ß-actin mRNA, in the midgut of the susceptible and Cry1Ac resistant larvae determined by real-time RT-PCR analysis. Error bars indicate standard errors of the means from analysis of 3 individuals.

By quantitative RT-PCR analysis, the mRNA levels of the cadherin gene, normalized to the β-actin mRNA as the internal control, in the midgut of the susceptible Cornell strain and the resistant backcross strain GLEN-Cry1Ac-BCS8 were determined to be similar without statistical difference (Figure 2).

### Cadherin in midgut of Cry1Ac-resistant Larvae is not Quantitatively Different from that in Susceptible Larvae

Quantitative proteomic analysis of *T. ni* midgut BBMV proteins by iTRAQ-2D-LC-MS/MS identified 1,464 proteins [35] and cadherin appeared to be a rare protein among the midgut BBMV proteins, accounting for 0.0014% (mol%) in contrast to the abundance of 0.42% (mol%) for APN1 (average of two sample sets) estimated by the formula: protein abundance (mol %)  =  emPAI of cadherin/∑(emPAI) ×100 [35], [49]. iTRAQ-2D-LC-MS/MS analysis of the midgut BBMV proteins from two sets of independent samples determined that for coverage of sample variations of 95% and 90% of the identified proteins the iTRAQ quantitative ratio [|log_2_ (115+117)/(114+116)|] cutoff point was 1.5 and 1.1, respectively [35]. The ratio of cadherin between the susceptible and resistant larvae in log_2_ was 0.28, or (cadherin in resistant larvae)/(cadherin in susceptible larvae) = 1.2. Therefore, there is no significant difference in abundance of cadherin in the midgut BBMVs between the susceptible and resistant larvae.

### Cadherin from Cry1Ac-resistant *T. ni* Larvae is not Different from the Susceptible Larvae in Binding with Cry1Ac by Ligand Blot Binding Analysis

**Figure 3:**
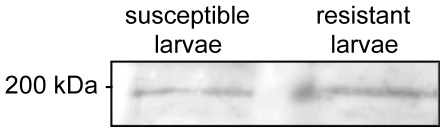
The 200 kDa Cry1Ac-binding cadherin was detected in midgut BBMV proteins from both susceptible and resistant *T. ni* by toxin overlay binding analysis.

We have previously reported that Cry1Ac bound to multiple protein bands, including the 200 kDa cadherin, from midgut BBMVs by toxin overlay binding analysis [35]. Our ligand blot binding assay in this study showed that the 200 kDa Cry1Ac-binding protein bands from the susceptible and resistant larvae were similar in staining intensity on the blot (Figure 3). Nano-LC-MS/MS analysis of the protein contents in the Cry1Ac-binding 200 kDa bands, excised from two adjacent lanes of SDS-PAGE gel loaded with equal amounts of BBMV proteins from the susceptible and the resistant larvae, respectively, identified the presence of cadherin in the protein bands and determined that the emPAI values of cadherin in the two excised gel slices from the susceptible and resistant larvae were similar (0.15 and 0.13, respectively), further indicating that the 200 kDa cadherin in the midgut BBMVs from resistant larvae was similar to that from the susceptible larvae in quantity and in binding with Cry1Ac.

## Discussion

High-level resistance of insects to Bt Cry toxins is commonly associated with alteration of midgut binding sites for the toxins [4], [50], [51]. Current understanding of the mode of action of Cry1A toxins suggests that the midgut cadherin serves as the binding protein in the first high-affinity binding event in the midgut-toxin interaction [13], [16], and mutations of the cadherin gene have been identified to be the genetic mechanism conferring high-level resistance to Cry1Ab or Cry1Ac in Lepidoptera [28]–[30]. The important role of cadherin in the mode of action of Cry1A toxins has been further implicated by rescued susceptibility in “Mode 1” type resistant insects with modified Cry1Ac which bypasses the interaction with cadherin in the pathogenesis of Bt toxins [52], [53]. In the case of Bt-resistance evolved in greenhouse populations of *T. ni,* high-affinity binding of Cry1Ac to midgut BBMVs from the Cry1Ac-resistant *T. ni* is not detectable [39]. However, the resistance is associated with down-regulation of APN1, and the resistance gene has been mapped at the ABCC2 locus [35], [37]. Therefore, the Cry1Ac-resistant *T. ni* from greenhouses is a unique biological system to examine if the “Mode 1” type Bt-resistance is universally conferred by mutations of the midgut cadherin gene or change of cadherin expression by the resistance-conferring *trans*-acting gene. Results from this study determined that the cadherin gene is not genetically linked with the resistance to Cry1Ac in *T. ni* (Table 2), demonstrating that mutations of the midgut cadherin gene is not the genetic basis for the “Mode 1” type resistance evolved in the greenhouse populations of *T. ni*. However, lack of genetic linkage of the cadherin gene with Cry1Ac does not exclude the possibility of alteration of cadherin by the resistance-conferring *trans*-regulatory mechanism. The results from quantitative analyses of the cadherin expression in this study confirmed that the midgut cadherin expression in the resistant *T. ni* larvae did not quantitatively differ from the susceptible *T. ni* at both mRNA (Figure 2) and protein (result from iTRAQ-2D-LC-MS/MS analysis) levels. Furthermore, the Cry1Ac toxin overlay binding assay functionally detected binding of Cry1Ac under an in vitro condition to the 200 kDa cadherin from the midgut BBMVs of both the susceptible and Cry1Ac-reistant *T. ni* (Figure 3). Moreover, nano-LC-MS/MS analysis of the 200 kDa Cry1Ac-binding protein excised from the SDS-PAGE gel in parallel with the toxin overlay binding assay further determined that the amount of cadherin in the 200 kDa Cry1Ac-binding band from midgut BBMVs of Cry1Ac-resistant *T. ni* larvae (emPAI = 0.13) was similar to that from the susceptible larvae (emPAI = 0.15). Therefore, the results from this study indicate that the “Mode 1” type resistance in *T. ni* is independent of alteration of the midgut cadherin.

The “Mode 1” type resistance in *T. ni*, as well as in *P. xylostella* and a strain of *Heliothis virescens*, has been recently identified to be associated with mutations of an ABC transporter gene, *ABCC2*, which is not in the same genetic linkage group as the other known Bt toxin binding protein genes [15], [37]. Whether *ABCC2* is the resistance-conferring gene and how the resistance-conferring *trans*-acting gene regulates the expression of the down-stream genes leading to loss of Cry1A binding sites in the midgut brush border remain unknown. The results from this study indicate that the Cry1Ac-resistance conferring *trans*-gene does not affect the midgut cadherin gene expression in *T. ni*.

The midgut cadherin, APNs and alkaline phosphatase are the major Cry1A toxin receptors identified in lepidopteran midgut [13]. More recently, ABCC2 has also been proposed to be a receptor [15]. The importance of cadherin as a receptor of the toxins for toxicity of Cry toxins has been well recognized and supported with experimental data. The midgut cadherin has been considered to be the primary midgut receptor for Cry1Ac and the GPI-anchored APN and alkaline phosphatase to serve as the secondary receptors [14], [16]. However, how cadherin is involved in binding of Cry toxins to the midgut brush border requires a better understanding. It has been reported that lack of midgut cadherin does not cause a detectable effect on Cry1Ac binding to the midgut BBMV from a strain of *H. virescens* (strain KCBhyb-RR) [54]. Similarly, Cry1Ac-resistance in a strain of *Pectinophora gossypiella* is associated with cadherin gene mutations, but the cadherin gene mutations do not affect the binding of Cry1Ac to the midgut BBMV [55]. In the greenhouse-derived Cry1Ac-resistant *T. ni* strain used in this study, the specific high-affinity binding of Cry1Ac to the midgut BBMV from this strain is not detectable [39], but the cadherin gene neither shows genetic linkage nor a change of expression at mRNA and protein levels (Table 2 and Fig. 2). However, expression of APN1 gene is significantly reduced to a level that the 110 kDa Cry1Ac-binding APN1 becomes absent [35]. The results that the cadherin was not altered at genomic, transcript and protein levels in Cry1Ac-resistant *T. ni* from this study and that the midgut BBMV from the resistant *T. ni* lacked binding affinity to the toxin from our previous study [39] are consistent with the finding that binding of Cry1Ac to midgut BBMV from a strain of *H. virescens* (KCBhyb-RR) does not change, even though the KCBhyb-RR strain lacks the midgut cadheirn [54]. Cadherin alone is not sufficient to constitute the measurable high-affinity binding sites for Cry1Ac and Cry1Ab in the midgut of *T. ni*.

For the interaction of Cry1A toxins with multiple midgut binding proteins, it has been proposed that binding of Cry1A toxin to the low-affinity but high-abundance APN to concentrate the toxin at midgut brush border occurs prior to binding to the high-affinity but low-abundance cadherin in the binding mechanism [13], which is supported with experimental data on differential binding of Cry1Ab mutants to the midgut receptors to become known as the “ping pong” binding mechanism [56]. The quantitative proteomic analysis of *T. ni* midgut BBMV proteins in this study confirmed that cadherin was a rare protein in the midgut BBMV, only accounting for 0.0014% (mol %) of the BBMV proteins, or 1/300 of APN1 in molar ratio. With the “ping pong” binding mechanism, interaction of the toxin with the cadherin could be facilitated by the abundant APN1.

Midgut cadherin from Lepidoptera has been shown to be the functional receptor for Cry1A toxin in insects by demonstration of acquired susceptibility of cultured insect cells to Cry1A toxins upon introduction and expression of a cadherin gene [18], [57], [58] and regions of putative toxin binding regions have been identified [57], [59]–[62]. Results from this study indicate that the cadherin in the Cry1Ac-resistant *T. ni* does not have any change in quantity in the midgut and its in vitro binding assayed by toxin overlay binding analysis is not different from the susceptible *T. ni*. However, the midgut BBMV from the resistant *T. ni* lacks high affinity specific binding sites for the toxin [39]. Therefore, binding of Bt toxins to isolated cadherin, such as the toxin overlay protein binding assay, is not necessarily indicative of the binding mechanism in insect midgut. The role of cadherin in binding with Cry toxins in insect midgut appears more complex and has yet to be understood.

## Supporting Information

Figure S1 **cDNA sequence and deduced amino acid sequence of** ***T. ni*** **midgut cadherin.** The 5732 bp cDNA contains an open reading frame of 5202 bp. The start codon ATG and the stop codon TAA are underlined and the PolyA signal sequence AATAAA is double-underlined. In the protein sequence, sequences for putative domains are shaded and indicated by domain names. Asterisks denote predicted putative N-glycosylation sites.
(DOC)
