# Supplementary material for: Resistance of Trichoplusia ni to Bacillus thuringiensis Toxin Cry1Ac Is Independent of Alteration of the Cadherin-Like Receptor for Cry Toxins
Source: PLoS One. 2012 May 14;7(5):e35991. doi: 10.1371/journal.pone.0035991 (PMC3351398; doi:10.1371/journal.pone.0035991)
Supplement: Figure S1 — cDNA sequence and deduced amino acid sequence of T. ni midgut cadherin. The 5732 bp cDNA contains an open reading frame of 5202 bp. The start codon ATG and the stop codon TAA are underlined and the PolyA signal sequence AATAAA is double-underlined. In the protein sequence, sequences for putative domains are shaded and indicated by domain names. Asterisks denote predicted putative N-glycosylation sites. (DOC) [file pone.0035991.s001.doc]

CCGAAGTTGTGTTGTTTGAGAAATATAATTATTTATTTTTTATTTAAATAATTCTCGTG 0059

TTCTGTGCAGTGTTGTATTGTATTGGATTAATTATAGCAAAGGTGCAGACATCATCTCCA 0119

TACTGACTCAGAAAATGTAAAATGGAGGCTGACGTCCGAATCACGACGGCAGCGCTGTTA 0179

M E A D V R I T T A A L L 0013

*Signal Peptide*

TTATTCGCTGCCAGCTTTGTCAACGCACAAAATGATGGATTGCGATGTACGTACATGAAA 0239

L F A A S F V N A Q N D G L R C T Y M K 0033

GAAATACCCAGAGGAGAAACTCCCGTTTTTGAAATAAAGGACTTTGATGGAGTACCATGG 0299

E I P R G E T P V F E I K D F D G V P W 0053

AACCAGCAGCCTCTTATACCACTGCCACAGCGAGAGGAACTGCGCATAGAAGATCCTGCC 0359

N Q Q P L I P L P Q R E E L R I E D P A 0073

TTTGCAGGAAATTCCATCGTCATGACAATTTTTATGGAGGAAGAGATCGAGGGAGAAATA 0419

F A G N S I V M T I F M E E E I E G E I 0093

GCTATAGCCAAGTTAAATTATAAAGGCACTGAAACCCCGAGCATCAGGCAACCCTTCGCA 0479

A I A K L N Y K G T E T P S I R Q P F A 0113

TCAGGTAGTTTCCACATGCTCGGTCCTGTCATTCGTCGGATTCCTGAAGATGGCGGCGAC 0539

S G S F H M L G P V I R R I P E D G G D 0133

TGGCACCTTGTTATCACTAATAAGCAGGACTACGAGGCTCCCGACATGCAGCGCTACTCG 0599

W H L V I T N K Q D Y E A P D M Q R Y S 0153

TTCGACATCTCGGTGCCGAGTGAATCAGCCGTCCTCATAGTGATGCTGGACATCATCAAC 0659

F D I S V P S E S A V L I V M L D I I N 0173

*Cadherin Repeat-1*

ATCGATGACAACGCTCCCATCATACACATGATCGACCGTTGCGAGATACCCGAGCCGGGC 0719

I D D N A P I I H M I D R C E I P E P G 0193

GAGTTAGGTCGCACGTCGTGTGTGTACACGGTGACGGACGCGGACGGTCGCCTCAGCACG 0779

E L G R T S C V Y T V T D A D G R L S T 0213

GAGTTCATGACGTATGAGATCGAGAGCGACCGCGACGACGCCGACTACTTCGAGCTGGTC 0839

E F M T Y E I E S D R D D A D Y F E L V 0233

*Cadherin Repeat-2*

AACGACCACACCATCGACCCTGACGACAAGACCACCCACATGGTCCTCTACCTACACAAA 0899

N D H T I D P D D K T T H M V L Y L H K 0253

GCCCTAGACTTCGAGCTCAATCCTCTTCATATATTCAGAGTCACGGCTTTGGACTCGAAG 0959

A L D F E L N P L H I F R V T A L D S K 0273

CCCAACACCCACACTGTGACGATGATGGTGCAAGTCCTAAACGTGGACCGCAGGAACCCG 1019

P N T H T V T M M V Q V L N V D R R N P 0293

CGCTGGCTGGACATCTTCGCCGTGCAGCAGTTCGATGAGAAGACTGTGCAGAGGTTCCAT 1079

R W L D I F A V Q Q F D E K T V Q R F H 0313

ATCAGAGCCATAGACGGTGACACGGGGCTCGACAGAGAAATCTACTATAAGCTGGAAGCG 1139

I R A I D G D T G L D R E I Y Y K L E A 0333

GACGAAGAAGATACATTCTTCTCCCTGGAGCCAATCGCGGGAGATCGCAGCGGCGCCACA 1199

D E E D T F F S L E P I A G D R S G A T 0353

*Cadherin Repeat-3*

TTAGTTGTTGACAAGATAGACAGAGACACTCTACAGCGGGAAGTGTTCCAGCTGTCCATA 1259

L V V D K I D R D T L Q R E V F Q L S I 0373

GTAGCGTATAAGTACGGTATCGATGATAAAGAGGGGAAGAACCCCTTCGAGACCAGAGCC 1319

V A Y K Y G I D D K E G K N P F E T R A 0393

AACATCGTGATCATCGTCAATGATGTCAACGATCAAAGGCCTCTCCCTTTCAAGAATACC 1379

N I V I I V N D V N D Q R P L P F K N T 0413

TACACAATAGAAATAGACGAAGAGACGCCCATGACCCTCAATTTAGAAGACTTTGGGTTC 1439

Y T I E I D E E T P M T L N L E D F G F 0433

CACGACATAGATCTCGGTGAAAACGCTCAATATGAAGTGTTCTTGGAGAGTGTATACCCT 1499

H D I D L G E N A Q Y E V F L E S V Y P 0453

*Cadherin Repeat-4*

GAAGGTGCCGAGGAAGCTTTCATGATCTCCCCGACGAGGGGATACCAGGAACAATCGTTC 1559

E G A E E A F M I S P T R G Y Q E Q S F 0473

ATAGTGTCGACGAGAAACCACCACCTTCTGGACTATGAAGTGGAAAAATATCAAAACATT 1619

I V S T R N H H L L D Y E V E K Y Q N I 0493

CAGCTAAAGGTAAGAGCAATAGACTTGAACGACACTCGTTTAACGGGCGAGGCGTTGTTG 1679

Q L K V R A I D L N* D T R L T G E A L L 0513

AACATTAACCTCCGGAACTGGAACGACGAGCTCCCGATCTTCGAGCACAGCGCGCAGACG 1739

N I N L R N W N D E L P I F E H S A Q T 0533

GTAGACTTTGACGAGACCGTCGGGAAGGACTTCCCTGTGGCCATCATCAAGGCTGACGAC 1799

V D F D E T V G K D F P V A I I K A D D 0553

AGAGATATCGGTGATAAAGTTGTACACTCATTGCTAGGCAATGCTGAAGATTATCTGACA 1859

R D I G D K V V H S L L G N A E D Y L T 0573

ATTGATCCGGACACTGGTGAGATATCCGTCGCTCACGATGACTACTTCGACTTCCATCGG 1919

I D P D T G E I S V A H D D Y F D F H R 0593

*Cadherin Repeat-5*

CAAAATGAGTTCTTTGTCCAGGTCCGCGCCACGGACACGCTGATGGAGCCCTACAACTCG 1979

Q N E F F V Q V R A T D T L M E P Y N S 0613

GTGACCGCGCAGCTCACCATCAGACTGCGGAACATCAACAACACGCCGCCCACGCTGCTA 2039

V T A Q L T I R L R N I N N T P P T L L 0633

CTCCCTCGCGGCAGTCCTGAAGTGGAAGAGAACGTGCCGCAAGACTTCGTGATACCGGCG 2099

L P R G S P E V E E N V P Q D F V I P A 0653

GAGATCGCGGCCACGGACCCCGACCTCGACGCACAACTGGAGTTCGAAATAGACTGGGAG 2159

E I A A T D P D L D A Q L E F E I D W E 0673

AGCTCGTACGCGACCAAGCAGGGAAGACCAGCTCCCGATGTTGAGTTCCATAAATGCGTG 2219

S S Y A T K Q G R P A P D V E F H K C V 0693

*Cadherin Repeat-6*

GAAATAATAACCATCCCCACGGAGACCCGTCACCGCGTCATCGGGCGCCTCGACGTGAGG 2279

E I I T I P T E T R H R V I G R L D V R 0713

ACCATCAGAGAGGGAGTCACCATCGACTACGAGGAGTTCGAGATCCTGTACCTCAGCATC 2339

T I R E G V T I D Y E E F E I L Y L S I 0733

AAGGTCTATGACAGGAATACTGTGGCTGGTGCTATCGATCATGCTGAATCGATCCTGGCC 2399

K V Y D R N T V A G A I D H A E S I L A 0753

ATCAACATAATCGACATGAACGACAACCCGCCGGTGTGGGCGGCGGGACAGCTGCGGCAG 2459

I N I I D M N D N P P V W A A G Q L R Q 0773

GCGCTGCGCGTGCGCGAGGGCTCTCCCGCCGGCGGGATCATCGGCTCACTGCTCGCCACC 2519

A L R V R E G S P A G G I I G S L L A T 0793

GACATCGACGGCCCGCTCTACAATAAAGTGCGGTACTCCATACATCCTAAGCCAGGCACC 2579

D I D G P L Y N K V R Y S I H P K P G T 0813

AAAGAAGGCCTAGTAGCGATCGATCCCATATTGGGTCAGCTGACGGTCCTGGGTGACGGA 2639

K E G L V A I D P I L G Q L T V L G D G 0833

GAGATAGACGCAGACGTGCCCAAGACCTGGACCCTGGAGTACACCGTCATCGCCAGCGAC 2699

E I D A D V P K T W T L E Y T V I A S D 0853

*Cadherin Repeat-7*

CGCTGTGTGGAGGACGACGGCGTGGCCTGCACCGGCACGGACCCCACTGTCTGGAACACC 2759

R C V E D D G V A C T G T D P T V W N T 0873

GAAGGCGATTTATCTATCGACATCATAGACACAAACAACAAGAACCCGGAGACCGCGAGC 2819

E G D L S I D I I D T N N K N P E T A S 0893

CCCAGCATTACCGTGTGGGTTTGGGAGAACGCGACCCATGGGGACCCCGTAGCACAGCTC 2879

P S I T V W V W E N* A T H G D P V A Q L 0913

TCCGCCACCGACCTTGACAGAGACGAGTTATACCACACGGTCCGCTACCAGATCCTGTAC 2939

S A T D L D R D E L Y H T V R Y Q I L Y 0933

TCGGTGAACCCGATGTTGCTGGAGCTGTTCGCGGTGGACCAGGACTCGGGCCTCATCACC 2999

S V N P M L L E L F A V D Q D S G L I T 0953

*Cadherin Repeat-8*

GTGCACTACACTAGCGACACGGTGCTGGACAGAGACGGCGACTATCCGGAACACACCATC 3059

V H Y T S D T V L D R D G D Y P E H T I 0973

TTCCTCAACCTCTTCGATAACTTCTTTTTCGATGGAGATGGGCAACGCAATATGGCGGAG 3119

F L N L F D N F F F D G D G Q R N M A E 0993

AAGAGAGTGCTCGTGGTTCTGTTAGATGTGAACGACAACGCGCCCGAACTGCCGCTACCT 3179

K R V L V V L L D V N D N A P E L P L P 1013

GAAGAACTGTCCTGGTCTGTGTCTGAAGACGAGAGAGAAGAAGTACGCGTACTACCACAT 3239

E E L S W S V S E D E R E E V R V L P H 1033

ATCTACGCTCCGGACAGAGACGAGCCGGACACGGATAACTCTAGGGTCGGCTATGCGATT 3299

I Y A P D R D E P D T D N S R V G Y A I 1053

*Cadherin Repeat-9*

CTCGGCCTTAAAGTGACCAACAGAGAGATCGAAGTCCCGGAGCTGTTCAACATGATTCAG 3359

L G L K V T N R E I E V P E L F N M I Q 1073

ATAGAGAACAAGACAGGAGAGCTCGAGACCGCTCGCCATCTGAAAGGATTCTGGGGAACT 3419

I E N* K T G E L E T A R H L K G F W G T 1093

TATAGTATACATATACAGGCGTACGACCACGGGATCCCTCAGCAGATATCTGAGGAGACG 3479

Y S I H I Q A Y D H G I P Q Q I S E E T 1113

TACACCCTCATCATCCGCCCTTACAACTACCACGAGCCGGTGTTCGTGTTCCCACAGGCT 3539

Y T L I I R P Y N Y H E P V F V F P Q A 1133

GGCAACACCTTCAGATTGTCCAGGGAGCAGTCGACAGTGAACGGCGTGTTGGTCCGCGTG 3599

G N T F R L S R E Q S T V N G V L V R V 1153

GACGGGCAGAGCTTCCCGCGCGTGTCGGCCACCGACGAGGACGGGCTGCACGCCGGCAGC 3659

D G Q S F P R V S A T D E D G L H A G S 1173

GTCAGCTTCAGCGTCGTCGGGGCTGCCGCTGAATACTTCTCCATGCGTAACTTCGAGGAC 3719

V S F S V V G A A A E Y F S M R N F E D 1193

*Cadherin Repeat-10*

AACACTGGCGAGCTTTACCTGTCGCAGCCCTTGCCTCTAGAAGACGATGGATTTGATATC 3779

N T G E L Y L S Q P L P L E D D G F D I 1213

ACCATCCGCGGTTCTGACGCGGGCACAGAGCCGGGCTCGCTGTTCTCGGAGGTTTCCTTC 3839

T I R G S D A G T E P G S L F S E V S F 1233

AGACTGGTGTTCGTGCCCACGCACGGGGACCCTGTCTTCAGCGTCAGCCAGTATACTGTC 3899

R L V F V P T H G D P V F S V S Q Y T V 1253

GCTTTTATAGAAAAAGAGGCTGGTTTACTGGAATCCCATCAACTGCCGAGAGCTGTGGAC 3959

A F I E K E A G L L E S H Q L P R A V D 1273

CCGAAGAACTACATGTGTGAGGAAATGAACGAGCCTTGTCATGAGATATACTACAGTATT 4019

P K N Y M C E E M N E P C H E I Y Y S I 1293

ATCGATAACAACGAGGAGGGTTACTTCCAAGTGGACTCGACCACAAACGTGATCTCCCTA 4079

I D N N E E G Y F Q V D S T T N V I S L 1313

*Cadherin Repeat-11*

TCCCGCGAGCTGGAGCGGGCCTCGCAGGCCAGTCACGTGGTCCGCGTGGCGGCCTCCAAC 4139

S R E L E R A S Q A S H V V R V A A S N 1333

ACTCTGCTGGACCCGGCCGCGCCGCCGCCGCTGCTGCCCTCCTCCACCTTCCTGCTCACC 4199

T L L D P A A P P P L L P S S T F L L T 1353

ATCAATGTCCGTGAGGCTGACCCGCGGCCAGTATTCGAGAGAGAGATCTACACCGCCGGC 4259

I N V R E A D P R P V F E R E I Y T A G 1373

ATCTACGAGACGGATACATCCAACAGGGAACTACTCACTGTTCATGCGACACATACCGAA 4319

I Y E T D T S N R E L L T V H A T H T E 1393

GGCCTAGACATAACGTACACGATGGACCTGGACACGATGGTGGTGGACCCCTCGCTGGAG 4379

G L D I T Y T M D L D T M V V D P S L E 1413

GGCGTGAGGGAGTCCGCCTTCACGCTGCACCCGAGCAGCGGCGTCCTGTCCCTCAACATG 4439

G V R E S A F T L H P S S G V L S L N M 1433

AACCCGCTCGACACCATGGTCGGCATGTTCGAGTTCGATGTCGTGGCTACAGACACCAGA 4499

N P L D T M V G M F E F D V V A T D T R 1453

*Membrane Proximal Region*

GGTGCAGAAGCCCGTACTGACGTGAAGATCTATCTGATCACTCATCTCAACAGAGTCTAC 4559

G A E A R T D V K I Y L I T H L N R V Y 1473

TTCTTGTTCAACAACACGCTGGATGTCGTTGACTCCAACAGAGCTTTCATAGCGGACACG 4619

F L F N* N T L D V V D S N R A F I A D T 1493

TTCTCGTCGGTGTTCAGCCTGACGTGCAACATCGACGCGGTGCTGCGCGCGCCGGACAGC 4679

F S S V F S L T C N I D A V L R A P D S 1513

AGCGGCGCCGCGCGCGACGACCGCACCGAGGTGCGCGCGCACTTCATACGGAACCACGTG 4739

S G A A R D D R T E V R A H F I R N H V 1533

CCCGCCACCACCGACGAGATAGAGCAGCTCCGTAGTAACACAATACTGCTGAGAGCGATT 4799

P A T T D E I E Q L R S N T I L L R A I 1553

CAGGAAACCCTGTTAACTCGCGAGCTGCATCTGGAGGACTTTGTGGGTGGCTCCAGCCCA 4859

Q E T L L T R E L H L E D F V G G S S P 1573

GAGCTGGGCGTGGACAACAGCCTCACAATATACGTGCTGGGCGCGCTCGCCGCGCTGCTC 4919

E L G V D N S L T I Y V L G A L A A L L 1593

*Transmembrane Domain*

GGCTTCCTGTGCGTGCTGCTACTCATCACCTTCATCGTCAGGACTAGAGCGCTCAACCGT 4979

G F L C V L L L I T F I V R T R A L N R 1613

CGCCTGGAAGCCCTATCCATGACTAAATACGGCTCAGTGGACTCCGGGCTGAACCGCGTG 5039

R L E A L S M T K Y G S V D S G L N R V 1633

GGTCTGGCCGCGCCCGGGACCAACAAGCACGCCGTCGAGGGCTCCAACCCCATCTGGAAC 5099

G L A A P G T N K H A V E G S N P I W N* 1653

GAGACCATCAAAGCGCCGGACTTCGATGCGATCAGCGACGTTTCTAACGACTCGGACCTG 5159

E T I K A P D F D A I S D V S N* D S D L 1673

*Cytoplasmic Domain*

ATCGGTATCGAGGACCTGCCGCAGTTCCGCAACGACTACTTCCCTCCCGCTGACGACAGC 5219

I G I E D L P Q F R N D Y F P P A D D S 1693

TCCCTAAGGGGCATCGTACTTGATAATCAGAACAACGACACGGTGGCGACTCACGGCAAC 5279

S L R G I V L D N Q N N* D T V A T H G N 1713

AACTTCAAGTTCAACGCCAGCCCCTTCAGCCCGGAGTTCGGGAACACGCCCATCCGGAGA 5339

N F K F N A S P F S P E F G N T P I R R 1733

TAAAACAAAATCTATGCGAAAGTAATCATGTAGTATAAGATACTGGCTGTTCCCTGTGGA 5399

AACTTGCCCAGAAGATTGTGGAAGAGAGACGCCGCTCTACGCCGTGTAGTGCCCTGTCCC 5459

GCTCCCACGTTAGGAAAACGGAAGGTCCACTGGACCTCCGCAATATTAAAACTATAACTG 5519

GGTAATTTTACTAAGGTATAACTTGGATAATCAGTCCAGTCGTTCATAAATTTTACCAAG 5579

TTTTTATACATATTATATAGATAGACATAGGTGATAATATTTAATATAATTGTGATGTCA 5639

AACTGTAGATATACTCGTAATATATAAGTGCATTTATTTTGTTATATACTGTATTTTAAT 5699

AAAAAATAAAAGATGAAAAAAAAAAAAAAAAAAAA 5734
